# Trends or evidence: What do orthodontists want to rely on?

**DOI:** 10.1590/2177-6709.24.3.007-008.edt

**Published:** 2019

**Authors:** Flavia Artese

**Affiliations:** 1 Universidade do Estado do Rio de Janeiro, Departamento de Odontologia Preventiva e Comunitária (Rio de Janeiro/RJ, Brazil).

The orthodontic world just recently came together at the AAO Annual Meeting, held in Los Angeles. As most professional conventions, it has the main purpose of offering to the attendee scientific, commercial and networking opportunities regarding the specialty. And it is quite natural that, while being exposed to all these resources, one ends up grasping the trend of the moment. 

It was quite evident that there has been a significant increase of interest in clear aligners and digital tools for clinical use. The amount of lectures and its attendance, as well as the large spectrum of different companies in the commercial exhibit with these products, have grown during the last years. And, for every new product in the market, this seems to be the general behaviour. It creates a new trend, that with time may or may not be truly incorporated into our clinical armamentarium.^1^

In the past, marketing for orthodontic products was targeted at the professional and his/her choice of using a new or specific product. Regarding clear aligners, the greatest shift I have observed in the commercial aspect is that, for the first time, to the best of my knowledge, an orthodontic product is being advertised directly to the public. Patients are being shifted to the category of consumers and being induced to demand a specific treatment modality, based on features such as more comfortable, faster and more predictable treatments.

Once a product is launched in the market, there is a lag between its clinical use and robust scientific evidence, and this is not an exception with clear aligners. Even though they have been in the market for almost 20 years, its clinical use is still having to rely on the professional’s clinical experience, the opinions of experts, and limited published evidence.^2^ ^,^ ^3^ The number of published papers on this subject has increased tremendously though. In a simple PubMed search, using the MeSH terms “clear aligners”, “orthodontic aligners”, there were 4 publications in 2003, after which the numbers have been increasing consistently, so that 33 articles were retrieved in 2018. However, most of these publications are mainly case reports, case series studies, or descriptions of the use of aligner systems.^3^ Publications on this topic are still expected to rise, since there are so many gaps, most specifically about predictability of the planned tooth movement.

So, what is available in the literature on predictability of treatment outcomes? A recent systematic review was published in 2017, which included 4 controlled clinical trials. Regarding treatment outcomes, it was found that the aligner group was less efficient than regular fixed appliances, but that evidence was yet insufficient.^2^ Another systematic review concluded that aligners are effective for anterior intrusion, posterior bucco-lingual inclinations and upper molar bodily movement, but ineffective for anterior extrusion, anterior bucco-lingual inclination and controlling rotation of teeth.^3^ Nevertheless, conclusions should be seen with caution due to the small number of studies available.

Despite this paucity of information in the literature, there are already claims that conventional fixed appliances will no longer be necessary,^4^ as if these two treatment modalities could never coexist or be used with the best of their qualities and outcomes, with the aim of providing our patients the best standard of care. In most new technology lectures that I attend today, it is quite common to be shown the diffusion of innovation curve proposed by Everett Rodgers. It seems to have replaced the pyramid of hierarchy of evidence, which was shown in most evidence-based lectures a few years ago. In this innovation curve, individuals are divided into innovators, early adopters, early majority, late majority and laggards, and it is believed that a new technology is finally adopted after it is self-sustained. Examples such as the shifts from analogic to digital photography or the direct purchase of air tickets rather than a travel agency are used, when comparing aligners to conventional orthodontic treatment. 

What really strikes my mind is that digital photography and online air tickets purchase were adopted innovations, along time, because they achieved predictable results. A digital photo camera would give you a photograph as good or better than the analogic one, and an online air ticket would allow you to acquire a ticket much faster and of your own choice. But regarding clear aligners, can we at this moment say the same for all treatment possibilities? Are we succumbing to the external pressure coming from direct consumer advertisement? Are we maintaining and guaranteeing to our patients our standards of treatment outcome? Or is this rush into being a full “adopter”, instead of a laggard, is affecting our clinical judgement and jeopardizing the chance of improving the adequate outcome of this orthodontic tool? After all the biggest trend I feel nowadays is the survival of our specialty as a health care profession based on evidence, instead of a cosmetic one based just on patient demands. 

Something worth thinking seriously about...

Good readings!

Flavia Artese - editor-in-chief (flaviaartese@gmail.com)
